# Evaluation of hematopoietic stem cell expansion in the presence of garcinol

**Published:** 2018

**Authors:** Azam Habibi, Masoud Soleimani, Amir Atashi, Mahshid AkhavanRahnama, Azadeh Anbarlou, Mansoureh Ajami, Monireh Ajami

**Affiliations:** 1 *Department of Hematology, Faculty of Medical Sciences, Tarbiat Modares University, Tehran, Iran *; 2 *Stem Cell and Tissue Engineering Research Center, Shahroud University of Medical Sciences, Shahroud, Iran*; 3 *Department of Applied Cell Sciences, School of Advanced Technologies in Medicine, Shahid Beheshti University of Medical Sciences, Tehran, Iran*

**Keywords:** Hematopoietic stem cells, Expansion, Garcinol, Small-molecule compounds

## Abstract

**Objective:**

The application of human cord blood (hCB) is limited to children by using relatively small volume of cord blood that does not contain enough hematopoietic stem cells (HSCs). So, efforts for applying cord blood stem cells in transplantation have led to establishment of some approaches for ex vivo expansion of HSCs such as garcinol.

**Materials and Methods:**

CD133+ HSCs were separated by a magnetic-activated cell sorting (MACS) system. Isolated cells were cultured with different doses of garcinol, SCF, TPO and FLT-3L. The optimal dose of garcinol for *ex vivo *expansion of HSCs was determined by direct counting. Flow cytometry was used to evaluate the expression of CD133 marker to check the ability of garcinol in maintenance of HSCs. Colony forming cell (CFC) assay was performed to evaluate clonogenic capability of treated cells. The level of expression of *CXCR4* gene was evaluated by RT-PCR. Data were analyzed using Student’s t test.

**Results:**

Our results showed that CD133^+^ HSCs in the presence of garcinol (5-10 µM) had high expansion activity and cell counting showed that the number of cells in treated group was higher than control group (1.9 –fold) and CFC assay showed that the number of colonies following treatment with garcinol had 1.3-fold increase. Treatment of HSCs with garcinol resulted in 9.6-fold increase in terms of *CXCR4* expression in comparison to control group.

**Conclusion:**

The present study showed that garcinol can improve *ex vivo *expansion of HSCs and enhance their potential for homing to bone marrow.

## Introduction

In recent years, autologous or allogeneic transplantation of hematopoietic stem cells (HSCs) has been performed as a medical approach for a number of diseases such as various blood diseases attributed to hematopoietic and immune dysfunctions as well as leukemias (Kondo et al., 2003 ▶; Verma et al., 2005 ▶).

Human cord blood is an unlimited and unique source of HSCs (Broxmeyer et al.,1989 ▶). Umbilical cord blood (UCB) transplants have been done worldwide in pediatric and adult patients (Gluckman et al., 2004 ▶). UCB stem cells are an interesting alternative to adult BM and mobilized peripheral blood (PB) stem cell. It has been shown that significant functional differences exist between UCB-derived HSCs and adult BM-derived HSCs. UCB-derived HSCs have a higher proliferation and expansion potential, which might be associated with their greater telomere length (Holyoake et al., 1999 ▶; Piacibello et al., 1997 ▶) and their ability to exit G0–G1 more rapidly (Mayani et al.,1998 ▶; Traycoff et al.,1994 ▶).

UCB-derived HSCs have been collected by a simple procedure with no danger for the mother or the newborn; in addition, these cells are less contaminated with blood-borne viruses than adult stem cell sources. The most important advantage of UCB is that there is no need to full human leukocyte antigen (HLA) compatibility and there is a lower risk of graft versus host disease (GvHD) in comparison to other sources because of low number of lymphocytes in UCB (Broxmeyer et al.,1989 ▶; Jaroscak et al.,2003 ▶; Wang et al.,1998 ▶).

However, a single UCB unit contains a lower number of HSCs as compared to other sources (Gluckman et al.,2004 ▶; Cohen et al.,2004 ▶). This is an important limiting factor when adult recipients undergo transplantation. To overcome this difficulty, various approaches have been used including transplantation of two UCB units (double umbilical cord blood transplantation, dUCBT) (Sideri et al.,2011 ▶), and intra-marrow injection (Frassoni et al.,2010 ▶) to improve the homing efficiency of the transplanted HSCs, or *ex vivo* expansion of HSCs (Jaroscak et al.,2003 ▶; De Lima et al.,2008 ▶; Kelly et al.,2009 ▶).

However, double cord transplantation might be associated with increased risk of GvHD and might not significantly reduce the time of neutrophil and platelet engraftment. In addition, intra-bone injection -although it was shown to be safe- did not shorten the time of neutrophil engraftment (Brunstein et al.,2009 ▶). For *ex vivo* expansion of HSCs, cell culture systems have used growth factors like stem cell factor (SCF), thrombopoietin (TPO), fms-like tyrosine kinase-3 ligand (FLT-3L), interleukin 6 (IL-6), Notch ligand Delta1, angiopoietin-like proteins, and pleiotrophin. However, protein-factor combinations have proven to be neither affordable nor readily available. Small-molecule compounds (SMCs) have been played vital roles in molecular biology and pharmaceutical therapy. The use of SMCs has also improved our understanding of signaling pathways that control stemness. Increasing the knowledge in this field can help researchers to promote current methods used for *ex vivo* expansion of HSCs (De Lima et al.,2008 ▶; Ding et al.,2004 ▶; Boitano et al.,2010 ▶).

Garcinol, a benzophenone derivative originally isolated from *Garcinia indica*, is one of the SMCs that can activate signaling pathways that promote HSC expansion (Krishnamurthy et al.,1981 ▶; Mantelingu et al.,2007 ▶). In this study, we evaluated the effect of garcinol on expansion of UCB-derived CD133^+^ HSCs. 

## Materials and Methods

**Preparation of the extracts**

Garcinol was obtained from Santa Cruz Biotechnology. Garcinol was stirred for 10 hr in DMSO at room temperature, and placed for 17 hr at 4°C. Then, the extract was filtered and stored at -20℃.

**Determination of the optimal dose of Garcinol**

To identify the optimal dose of garcinol that exerts the optimum effect on expansion of HSCs. CD133^+^ HSCs were cultured with different doses of garcinol in the presence of SCF, TPO and FLT-3L (Stem Cell Technology). After 8 days, cells were stained with trypan blue and viable cells were counted.

**Mononuclear cells separation and cell culture**

CD133^+^ HSCs were separated from human cord blood (hCB) obtained from the Iranian Blood Transfusion Organization. Mononuclear cells were separated by Ficoll-Paque PLUS (GE Healthcare) density gradient centrifugation. CD133^+^ HSCs were isolated using standard enrichment procedures (MACS indirect CD133 MicroBead Kit (human-Miltenyi Biotec). Purified CD133^+^ HSCs were used immediately for experiments. hCB-derived CD133^+^ HSCs were plated at 1×10^4^ cells/well in a 48-well plate and cultured in serum-free medium, StemspanTM H3000, supplemented with a 1% penicillin-streptomycin mixture (Sigma) at 37°C with 5% CO_2_. Recombinant human SCF at 100 ng/ml, FLT-3L at 50 ng/mL, TPO at 20 ng/ml, and 10 µm garcinol were added to each well.

**Flow cytometry**

The percentage of CD133^+^ HSCs was assayed by flow cytometry (ATTUNE^®^ Flow cytometer (Applied Biosystems), Stem Cell Technology Research Center, Tehran, Iran) on days 0, 6 and 11. Treated and non-treated HSCs were incubated with phycoerythrin (PE)-conjugated anti-CD133 antibodies (Miltenyi Biotec) according to the manufacturer’s instructions. 

**Colony forming cell (CFC) assay**

The potential of HSCs to proliferate and differentiate into colonies was evaluated before and after expansion with garcinol by colony forming cell (CFC) assay. About 1000-1500 cells were mixed in 2 ml of methocult H4435 (Stem Cell Technology) containing 50 ng/mL human SCF, 20 ng/mL FLT-3L, and 20 ng/ml TPO. The number of colonies which were incubated at 37 °C for 14 days, was counted by using an inverted microscope.

**Real-time reverse transcription PCR and Gene expression analysis**

UCB-derived CD133^+^ HSCs were isolated by cell sorting with MACS. CD133^+^ HSCs were cultured in the presence of garcinol or DMSO for 11 days. Total RNA of the isolated cells was extracted on days 0, 6, and 11. Total RNA extraction, cDNA synthesis, real-time PCR and calculation of gene expression were performed as previously described (Anbarlou et al.,2015 ▶). The PCR primer sequences used for real-time PCR in this study, are shown in Table 1.

**Statistical analysis**

All tests and analyses were conducted in triplicate. Data means were compared using Student’s t test. Statistical analyses were performed by SPSS version 16.0 and GraphPad Software (GraphPad PRISM V 5.0).

## Results

**Determination of the optimal dose of garcinol**

**Figure 1:**
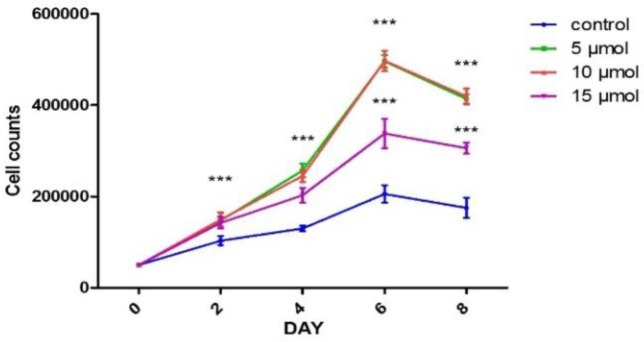
Determination of the optimal dose of garcinol. The results demonstrated that the best expansion activity of HSCs was achieved in the presence of 5 or 10 µM of garcinol. (Mean±SEM, n=3). *p<0.05, **p<0.01, and *** p<0.001

CD133^+^ HSCs were treated with different doses of garcinol in the presence of SCF, TPO, and FLT-3L for 8 days to find the optimal dose of garcinol that has the best effect on HSCs expansion. The results showed that CD133^+^ HSCs in the presence of garcinol 5-10 µM had high expansion activity (Figure 1). In accordance with the study of Taito Nishino et al. (2011) ▶, the 10µM concentration of garcinol was selected for the experiments.

**Garcinol improves the expansion of human hematopoietic Stem Cell**

**Figure 2:**
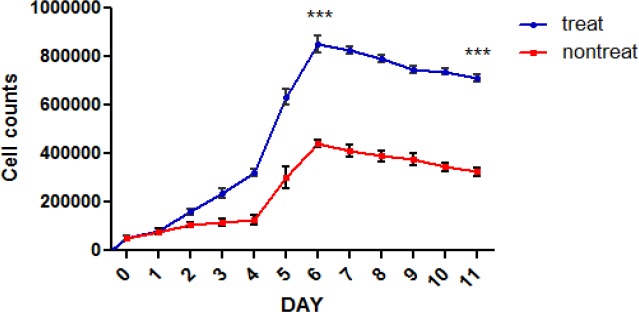
Garcinol effect of the expansion of human hematopoietic stem cells. Garcinol efficiently increased the numbers of HSCs. Bar graph indicated total cell counts (mean±SEM, n=3). *p<0.05, **p<0.01, and *** p<0.001

To recognize biologically-active natural products that affect *ex vivo* expansion of HSCs, hCB-derived CD133^+^ HSCs were cultured with garcinol (10 µM) in the presence of SCF, TPO, and FLT-3L. After 11 days, the number of CD133^+^ HSCs was enumerated (Figure 2). Cell counting showed that the number of cells in treated group was higher than control group (1.9 –fold) (control cells were treated with DMSO to remove the possible effect of drug solvent). 

**Flow cytometry results**

**Figure 3:**
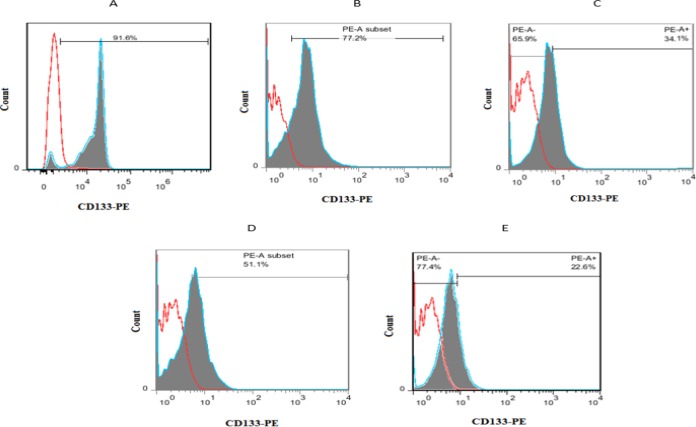
Flow cytometry results. Reported data (A-E) is one of our repetitions (n=3). Purity of CD133+ HSCs on Day 0 (A); Percentage of CD133+ HSCs in cells treated with garcinol on days 6 (B) and day 11 (C); Percentage of CD133+ HSCs in control group on days 6 (D) and 11 (E)

**Figure 4:**
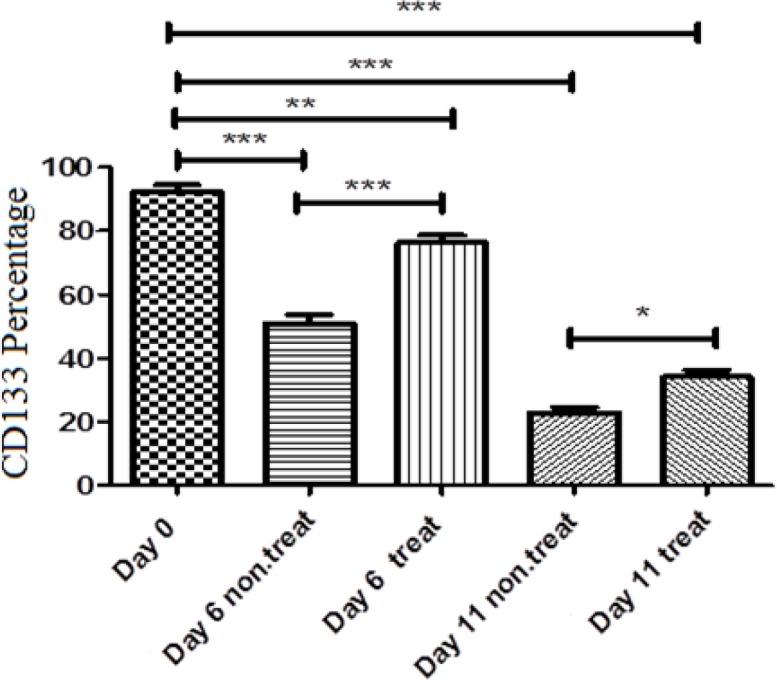
Percentage of CD133^+^ HSCs. Graph shows percentage of CD133^+^ HSCs on days 0, 6 and 11 after treatment with garcinol and control group in 3 repetitions. Values are shown as mean±SEM. *p<0.05, **p<0.01, and *** p<0.001

The purity of CD133^+^ HSCs was evaluated by flow cytometry on days 0, 6 and 11. The percentage of CD133^+^ cells was nearly 92% after immunomagnetic separation (Figure 3). The percentage of CD133 marker in HSCs in control group was 50% and 23% on days 6 and 11, respectively and in treated group, it was 76% and 36% on days 6 and 11, respectively (Figure 4).

**Table 1:** The PCR primers sequences used for quantitative real-time PCR

**Gene**	**Sequence**
*CXCR4 F* *CXCR4 R* *GAPDH F* *GAPDH R*	CGC CAC CAA CAG TCA GAG AAC ACA ACC ACC CAC AAG TC CCT CAA GAT CAT CAG CAA TG CAT CAC GCC ACA GTT TCC

**Hematopoietic colony forming cell assay**

**Figure 5:**
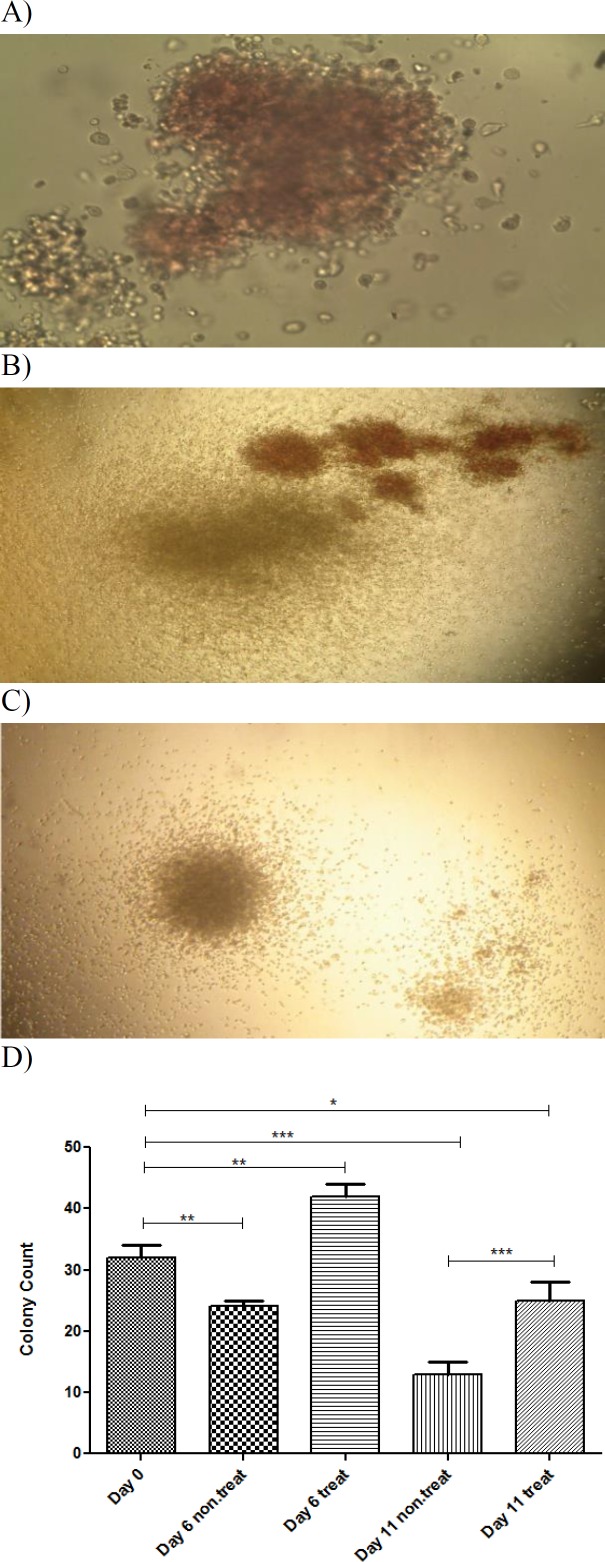
Hematopoietic colony forming cell assay. Numbers of colony forming units in different groups after 14 days. (A) Erythroid colony; (B) CFU- Mix; (C) CFU-Granulocyte-Macrophage (CFU-GM); Magnification X100. (D) Bar graph shows the number of colonies in different groups; Values are shown as mean±SEM. *p<0.05, **p<0.01, and ***p<0.001 show statistically significant differences

Colony forming cell (CFC) assay showed that the number of colonies in cells treated with garcinol increased (on average 1.3-fold; p=0.01) (Figure 5). These data demonstrated that garcinol can promote colonogenic capacity of HSCs. When cells were separated from body, they had started asymmetrical division *in vitro*. Thus, some of them had been differentiated into progenitor cells which decreased their colonogenic capacity. So, the number, percentage and the colonies of the untreated cells reduced by increasing time. Garcinol could partially prevent this outcome.

**Effects of garcinol on** ***CXCR4*** **gene expression**

**Figure 6:**
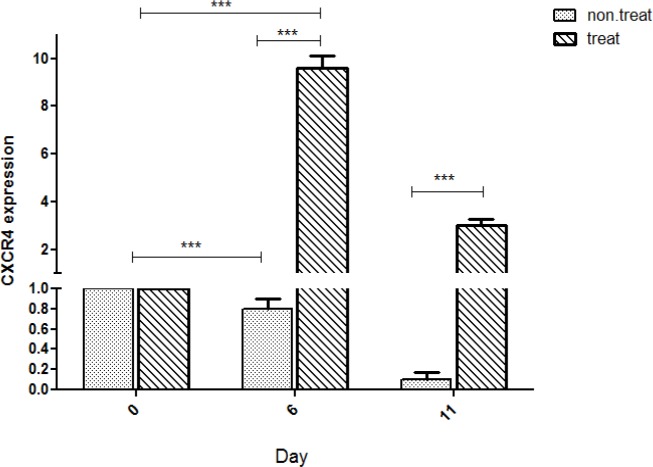
Effects of garcinol on *CXCR4* expression. Bar graph shows relative expression levels of *CXCR4* gene. The relative expression levels of *CXCR4* in HSCs treated with garcinol was increased (9.6-fold; p<0.001) in comparison to control group; Values are shown as mean±SEM. *p<0.05, **p<0.01, and ***p<0.001 show statistically significant differences

CXCR4, a chemokine receptor, has an important role in quiescence and homing of HSCs. Treatment of HSCs with garcinol for 11 days led to up-regulation of *CXCR4* in cells. Expression levels of *CXCR4* were measured by qRT-PCR. Treatment of HSCs with garcinol resulted in 9.6-fold increase in *CXCR4* expression in comparison to control group (Figure 6).

## Discussion

Our results showed that garcinol (10 µM) markedly increased the expansion of HSCs as compared to control. In addition, colony formation assay showed 1.3-fold increase in colony formation in HSCs treated with garcinol. Treatment of HSCs with garcinol resulted in 9.6-fold increase in *CXCR4* expression in comparison to control group. Furthermore, garcinol may enhance HSCs homing by up-regulating the expression of *CXCR4* gene. Garcinol or camboginol, is a poly isoprenylated benzophenone derivative which is isolated from *Garcinia indica* (Krishnamurthy et al.,1981 ▶).

Garcinol was applied for *ex vivo* expansion of HSCs because of its ability in inhibition of histone acetyltransferases (HAT 300). Also, garcinol reduces the level of p53 acetylation. p53 limits the self-renewal of HSCs (TeKippe et al., 2003 ▶). Increased expression of *Bmi-1* promotes HSCs self-renewal. Overexpression of *Bmi-1* enhances symmetrical division of HSCs by increasing the transcriptional repression of tumor suppressor genes, such as *Ink4a* and *Arf* (Iwama et al., 2004 ▶).

Garcinol has several biological activities, specifically anticancer potential which is mediated via its anti-oxidative, anti-inflammatory, anti-angiogenic, and pro-apoptotic activities. The beneficial properties of garcinol are dose-dependent (Saadat et al.,2012 ▶). There are few articles that have focused on the effect of garcinol on *ex vivo *expansion of HSCs. First, Taito Nishino et al. (2011) ▶ introduced garcinol as a novel stimulator of hCB-derived HSCs expansion (Nishino et al., 2011 ▶). HSCs can be isolated based on CD34 or CD133 cell surface markers. Early primitive HSCs do not express CD34 marker, so we applied CD133 marker for isolation of HSCs (Mossahebi et al., 2017 ▶). Taito Nishino et al. (2011) ▶ isolated HSCs by using anti- CD34 antibody. They reported that garcinol (10 µM) increased the numbers of CD34^+^CD38^– ^HSCs by more than 4.5-folds (Nishino et al., 2011 ▶), while in the current study the number of CD133^+^ cells were increased by more than 1.9- folds. They only evaluated the expansion of HSCs and did not address the engraftment potential of expanded cells. 

It is thought that garcinol acts in a similar way to that of Bmi1 by inhibiting the activity of histone acetyltransferases (Konuma et al.,2010 ▶). In addition to garcinol, other SMCs have been found to be effective on HSCs expansion by inhibiting components of the epigenetic machinery (Nishino et al.,2012 ▶; Ko et al.,2011 ▶). It seems that an important limitation of garcinol usage is the close proximity of toxic doses and non-toxic efficient doses. More studies in animals will certainly reveal the quality of expanded cells. 

The present study showed that garcinol can improve *ex vivo *expansion of HSCs and enhance their potential for homing to bone marrow. We suggest that a combination of SMCs with different methods may improve the efficacy of current methods for *ex vivo* expansion of HSCs.

## References

[B1] Anbarlou A, Atashi A, Soleimani M, AkhavanRahnama M, Bohloli M, Mossahebi-Mohammadi M (2015). Differential characteristics of CD133+ and CD133− Jurkat cells. In Vitro Cell Dev Biol Anim.

[B2] Boitano A, Wang J, Romeo R, Bouchez L, Parker A, Sutton S, Denison M (2010). Aryl hydrocarbon receptor antagonists promote the expansion of human hematopoietic stem cells. Science.

[B3] Broxmeyer H, Douglas G, Hangoc G, Cooper S, Bard J, English D, Boyse E (1989). Human umbilical cord blood as a potential source of transplantable hematopoietic stem/progenitor cells. Proc Natl Acad Sci.

[B4] Brunstein C, Barker J, Weisdorf D, Defor T, McKenna D, Chong S, Wagner J (2009). Intra-BM injection to enhance engraftment after myeloablative umbilical cord blood transplantation with two partially HLA-matched units. Bone Marrow Transplant.

[B5] Cohen Y, Nagler A ((2004)). Umbilical cord blood transplantation–how, when and for whom?. Blood Rev.

[B6] De Lima M, McMannis J, Gee A, Komanduri K, Couriel D, Andersson B, Champlin R (2008). Transplantation of ex vivo expanded cord blood cells using the copper chelator tetraethylenepentamine: a phase I/II clinical trial. Bone Marrow Transplant.

[B7] Ding S, Schultz P (2004). A role for chemistry in stem cell biology. Nat Biotechnol.

[B8] Frassoni F, Varaldo R, Gualandi F, Bacigalupo A, Sambuceti G, Sacchi N, Podestà M (2010). The intra-bone marrow injection of cord blood cells extends the possibility of transplantation to the majority of patients with malignant hematopoietic diseases. Best Pract Res Cl Ha.

[B9] Gluckman E (2004). Ex vivo expansion of cord blood cells. Exp Hematol.

[B10] Holyoake T, Nicolini F, Eaves C (1999). Functional differences between transplantable human hematopoietic stem cells from fetal liver, cord blood, and adult marrow. Exp Hematol.

[B11] Iwama A, Oguro H, Negishi M, Kato Y, Morita Y, Tsukui H, Koseki H (2004). Enhanced self-renewal of hematopoietic stem cells mediated by the polycomb gene product Bmi-1. Immunity.

[B12] Jaroscak J, Goltry K, Smith A, Waters-Pick B, Martin P, Driscoll T, Burhop S (2003). Augmentation of umbilical cord blood (UCB) transplantation with ex vivo–expanded UCB cells: results of a phase 1 trial using the AastromReplicell System. Blood.

[B13] Kelly S, Sola C, De Lima M, Shpall E (2009). Ex vivo expansion of cord blood. Bone Marrow Transplant.

[B14] Ko K, Holmes T, Palladinetti P, Song E, Nordon R, O’Brien T, Dolnikov A (2011). GSK‐3β Inhibition Promotes Engraftment of Ex Vivo‐Expanded Hematopoietic Stem Cells and Modulates Gene Expression. Stem Cells.

[B15] Kondo M, Wagers AJ, Manz M, Prohaska S, Scherer D, Beilhack G, Weissman I (2003). Biology of hematopoietic stem cells and progenitors: implications for clinical application. Annu Rev Immunol.

[B16] Konuma T, Oguro H, Iwama A (2010). Role of the polycomb group proteins in hematopoietic stem cells. Dev Growth Differ.

[B17] Krishnamurthy N, Lewis Y, Ravindranath B (1981). On the structures of garcinol, isogarcinol and camboginol. Tetrahedron Lett.

[B18] Mantelingu K, Reddy B, Swaminathan V, Kishore A, Siddappa N, Kumar G, Sadhale P (2007). Specific inhibition of p300-HAT alters global gene expression and represses HIV replication. Chem Biol.

[B19] Mayani H, Lansdorp P (1998). Biology of human umbilical cord blood‐derived hematopoietic stem/progenitor cells. Stem Cells.

[B20] Mossahebi-Mohammadi M, Atashi A, Kaviani S, Soleimani M (2017). Efficient expansion of SALL4–transduced umbilical cord blood derived CD133+ hematopoietic stem cells. Acta Med Iran.

[B21] Nishino T, Miyaji K, Ishiwata N, Arai K, Yui M, Asai Y, Iwama A (2009). Ex vivo expansion of human hematopoietic stem cells by a small-molecule agonist of c-MPL. Exp Hematol.

[B22] Nishino T, Wang C, Mochizuki-Kashio M, Osawa M, Nakauchi H, Iwama A (2011). Ex vivo expansion of human hematopoietic stem cells by garcinol, a potent inhibitor of histone acetyltransferase. PLoS ONE.

[B23] Nishino T, Osawa M, Iwama A (2012). New approaches to expand hematopoietic stem and progenitor cells. Expert Opin Biol Ther.

[B24] Piacibello W, Sanavio F, Garetto L, Severino A, Bergandi D, Ferrario J, Aglietta M (1997). Extensive amplification and self-renewal of human primitive hematopoietic stem cells from cord blood. Blood.

[B25] Saadat N, Gupta S (2012). Potential role of garcinol as an anticancer agent. J Oncol.

[B26] Sideri A, Neokleous N, De La Grange PB, Guerton B, Kerdilles M, Uzan G, Gluckman E (2011). An overview of the progress on double umbilical cord blood transplantation. Haematologica.

[B27] TeKippe M, Harrison DE, Chen J (2003). Expansion of hematopoietic stem cell phenotype and activity in Trp53-null mice. Exp Hematol.

[B28] Traycoff CM, Abboud MR, Laver J, Clapp DW, Srour EF (1994). Rapid exit from G0/G1 phases of cell cycle in response to stem cell factor confers on umbilical cord blood CD34+ cells an enhanced ex vivo expansion potential. Exp Hematol.

[B29] Verma IM, Weitzman MD (2005). Gene therapy: twenty-first century medicine. Annu Rev Biochem.

[B30] Wang XN, Sviland L, Ademokun AJ, Dunn J, Cavanagh G, Proctor SJ, Dickinson AM (1998). Cellular Alloreactivity of Human Cord Blood Cells Detected By T-Cell Frequency Analysis and A Human Skin Explant Model1. Transplantation.

